# Development and validation of a predictive model for depression risk in older patients with multiple chronic diseases in the community: cross-sectional study based on a health ecology model

**DOI:** 10.3389/fpubh.2026.1733851

**Published:** 2026-03-24

**Authors:** Zhirong Xu, Wen Ding, Jing Zhao, Guolian Liu, Hui Wan

**Affiliations:** 1 Department of Urology, General Hospital of Ningxia Medical University, Yinchuan, China; 2 Nursing Department, General Hospital of Ningxia Medical University, Yinchuan, China; 3 School of Nursing, Ningxia Medical University, Yinchuan, China

**Keywords:** comorbidity, depression, older patients, health ecology model, nomogram, risk prediction model

## Abstract

**Aim:**

Guided by the health ecology model, which posits that an individual’s health is shaped by the dynamic interplay between personal and environmental factors, we investigated factors associated with depression in community-dwelling older adults with multimorbidity and developed a nomogram-based risk prediction model. While previous research has predominantly focused on depression in the context of single chronic diseases, the psychosocial and clinical complexities inherent to multimorbidity remain largely overlooked. This study addresses this gap by constructing a tailored prediction model that integrates the multidimensional determinants of depression in this vulnerable population.

**Methods:**

Using convenience sampling, a questionnaire survey was conducted among 679 older patients with chronic diseases from 12 community health institutions in China between March and August 2023. Participants were randomly divided into a training group (*n* = 475, 70%) and a validation group (*n* = 204, 30%). Depressive status was assessed using the Geriatric Depression Scale-15 (GDS-15). Logistic regression analysis identified factors associated with depression, based on which a nomogram prediction model was constructed. The model was internally validated using the Bootstrap method with 1,000 resamples. Its predictive performance was comprehensively evaluated using receiver operating characteristic (ROC) curve analysis, calibration curves, and decision curve analysis.

**Results:**

The prevalence rate of depression among older community residents with comorbid chronic conditions was 29.90%. We identified specific predictors for depression in this population: age ≥ 80 years, excess body weight, types of medication, self-management (the ability to actively manage one’s health conditions), self-efficacy (confidence in one’s ability to perform health-related actions), and educational level. For the training group, the area under the receiver operating characteristic (ROC) curve was 0.815, indicating a model accuracy of 74%, a sensitivity of 79%, and a specificity of 72%. Hosmer-Lemeshow fitting testing results in a χ^2^ value of 8.801 (*p* = 0.359).

**Conclusion:**

Our new predictive model for the risk of depression in older patients in the community with multiple chronic diseases exhibited good discrimination, calibration and clinical practicability, serving as a valuable reference for the early detection of depression among this population.

## 1 Introduction

Depression is a common psychological disorder that occurs in individuals relating to emotion, cognition and behavior, predominantly manifesting as persistent emotional sadness, loss of interest, memory decline, social avoidance and a reduced sense of self-worth (1, 2). The Global Burden of Disease Study reported that depression ranks 13th globally in terms of disease burden and ranks first in terms of the burden generated by mental disorders (3). According to statistics, approximately 260 million people worldwide suffer from depression; of these, Chinese individuals account for 21.3% (4). By 2030, the global population aged ≥60 years is projected to reach 1.4 billion, accounting for 16.5% of the total population. By 2050, this number is expected to more than double, exceeding 2.1 billion and representing over 22% of the world’s population (5). Due to a decline in their own physical functionality, the accumulation of diseases, and changes in lifestyle, older adults are more prone to depressive symptoms than other age groups, and can influence their sleep, daily activities, cognitive functions and even safety (6–10). Previous studies have reported a wide variation in the prevalence of depression among older adults, ranging from 7.7 to 81.1%, which may be attributed to differences in study populations, assessment tools, diagnostic criteria, and research settings (11). Consequently, depression has become a significant public health challenge that cannot be ignored.

Over recent years, older patients with multiple chronic diseases have become increasingly common. In developed countries, approximately three quarters of older adults are also affected by a variety of chronic diseases (12); furthermore, the incidence of older adults with multiple chronic diseases in China has reached 44.9% (13). Studies have shown that depression is a risk factor for cardiovascular diseases, thus increasing the risk of chronic diseases such as hypertension, coronary heart disease and diabetes (13). Furthermore, the incidence of depression is significantly elevated among older patients with multiple chronic diseases when compared to their counterparts who are not affected by such diseases (14). A previous survey of the Guangdong Province community in China revealed that older patients with three chronic diseases faced a significantly elevated risk of depression when compared to those with two diseases (15). Moreover, the mechanism of interaction between multiple chronic diseases and depression is known to be complex, diverse, and affected by numerous physio-psycho-social factors, including polypharmacy, living alone, social support, and economic pressure (16, 17). However, existing studies of depression in older patients with multiple chronic diseases mostly investigated the influential factors from a single dimension (18, 19), lacked exploration of multi-faceted factors and were unable to predict the risk of depression occurrence in an accurate manner.

The health ecological model (HEM) regards an individual’s health as a complex system interacting with the environment, emphasizing that health is comprehensively influenced by multiple factors and multiple layers, involving personal traits, behavior characteristics, interpersonal network, living and working conditions and policy environment (20). The application of this model is conducive to investigating the factors that might influence the occurrence of depression in patients from multiple dimensions and perspectives of physiology, psychology and society. Many researchers have used this model to investigate the factors that could influence depression in middle-aged and older patients with multiple chronic diseases and older female patients; these studies have demonstrated that both individual and environmental factors can influence depression in these patient cohorts (21, 22).

Compared with the investigation of correlations between variables via the analysis of influencing factors, a risk prediction model can integrate multiple factors to construct a specific model and then generate individualized probabilistic risk assessment. Such models can not only predict the individual risk of developing depression but also identify the main factors affecting the risk probability, thereby assisting medical staff in formulating practical risk management strategies for depression (23, 24). Nomograms, as tools for visual prediction models, integrate multiple influencing factors and use visual line segments to express the inter-relationships between various variables in a prediction model based on the extent to which each influencing factor can contribute to the outcome variables (25). This strategy is advantageous because it exhibits calibration and clinical practicability, and can predict future events in a relatively accurate manner (26), thereby assisting medical staff to rapidly acquire individual risk values through point-line correspondence; this allows them to make better clinical decisions. Therefore, under the guidance of the HEM, the analysis of a risk model for depression in older patients with multiple chronic diseases could reveal the risk of depression from multiple dimensions and in a more accurate manner than existing methods.

Under a background of limited medical resources, communities have become the main living and rehabilitation space for older patients with comorbidities. Community-based research is motivated not only by resource constraints but also by alignment with key public health priorities, including accessibility, preventive orientation, population representativeness, and ecological validity. However, previous studies relating to the risk of depression in older adults with multi-morbidity have mostly focused on hospitalized patients (27), with insufficient attention paid to older patients with multiple chronic disease in the community. Therefore, the core goal of this study was to construct and verify a predictive model for the risk of depression in community-dwelling older patients with multiple chronic diseases based on the HEM. Our aim was to provide a reference for the early identification of depression and the formulation of precise intervention strategies in older adults with multiple chronic conditions living in the community.

## 2 Methods

### 2.1 Design and participants

From March to August 2023, we used a convenience sampling method to recruit older adults with multimorbidity as the survey participants in 12 community health service institutions in China. The inclusion criteria were as follows: permanent residents of the community aged ≥ 60 years (local residence time ≥ 6 months); diagnosed with two or more chronic diseases (including hypertension, osteoarthropathy or rheumatism, diseases of the digestive system, heart disease, chronic obstructive pulmonary disease, diabetes, stroke, chronic bronchitis, fatty liver, cervical/lumbar spine diseases) by a hospital of Grade II Class A or above; clear consciousness and no communication barriers; provided informed consent and participated in this research on a voluntary basis. The exclusion criteria were as follows: end-stage disease of the vital organs, including the heart, liver and lungs or patients receiving palliative care; and patients who were already participating in relevant experimental studies.

### 2.2 The selection of independent variables

The selection of independent variables was based on a review of the relevant literature and classified according to the five levels of the health ecology model, as detailed in Table 1. It should be noted that, due to data accessibility constraints inherent in community-based cross-sectional studies, the present model has certain limitations in variable selection at higher ecological levels, particularly within the “policy environment” dimension. For example, it does not directly assess the physical accessibility of community health resources, the built environment of communities, or specific aspects of mental health policies. These factors generally require precise evaluation through environmental audits, geographic information systems, or policy text analysis. Therefore, at higher ecological levels, this study primarily employed proxy variables that could be effectively collected through individual questionnaires, such as “type of health insurance,” to approximate the ecological perspective as closely as possible under the existing conditions.

**Table 1:** Selection of independent variables.

Hierarchy layer	Connotation	Independent variables
Personal traits	The inherent and relatively stable physiological and disease characteristics of an individual.	Gender, age, body mass index (BMI), BMI, the number of chronic diseases, family history
Behavior characteristics	Individual modifiable health-related behaviors and cognitive processes.	Smoking, drinking, the number of physical exercises per week, regular medication, type of medication, self-management, self-efficacy
Interpersonal network	The resources and pressures provided by an individual’s direct social relationships and environment.	Social support, marital status, living conditions
Living and working conditions	The social economic environment and material living conditions of individuals.	Education, working status, occupation, family per capita income, address
Policy environment	Macro institutional factors affecting the accessibility and quality of medical and health services.	Medical payment methods

### 2.3 Measuring tools

The questionnaire used to acquire basic information was designed by the researchers in an independent manner to collect two aspects of information from the participants: basic information such as gender, age, education level, and marital status; along with disease-related information, including disease name, disease duration, and the number of comorbidities. Age was categorized into three groups (60~, 70~, and ≥80 years), with the 60 ~ year group as the reference. According to the Guidelines for the Prevention and Control of Overweight and Obesity in Chinese Adults, BMI was categorized as follows: BMI < 18.5, underweight; 18.5 ≤ BMI < 24.0, normal weight; 24.0 ≤ BMI < 28.0, overweight; BMI ≥ 28.0, obesity. Educational level was classified into four categories in accordance with China’s national education system: primary school and below, junior high school, senior high school/college, and bachelor’s degree and above. Med types referred to the number of medications currently used by the study participants.

The Social Support Rating Scale (SSRS) was first reported by Xiao et al. (28), consisting of three dimensions: objective support, subjective support and support utilization degree, with a total of 10 items. The score for each item ranges from 1 to 4, with a total of 12 items. The score is positively correlated with the level of social support. A score < 33 is considered low, a score between 33 and 45 is considered medium, and a score > 45 is considered high. The Cronbach’s *α* coefficient for this scale is 0.90 (The Cronbach’s α of the modified scale was greater than 0.7, which was considered to have good reliability).

The Chronic Disease Self-Management Scale (CDSMS) was first developed by Dongbo et al. (29) and includes three dimensions: physical exercise (6 items), cognitive symptom management practice (6 items), and communication with doctors (3 items). We also used a Likert 6-level scoring system with a total score ranging from 0 to 69 points. The higher the score, the better the self-management behavior; the Cronbach’s *α* coefficient for this scale is 0.93.

The Chronic Disease Self-efficacy Scale (CDSES) was first developed in 2001 by the Chronic Disease Research Center in the United States (30). This scale consists of two dimensions: symptom management and disease commonality management, with a total of six items. The score for each item ranges from 1 to 10, indicating ‘no confidence at all’ to ‘very confident’, respectively. The total score for this scale is the average score of the 6 items. The minimum score was 1 and the maximum score was 10. A score > 7 points indicates a high level of self-efficacy, 5 to 7 points indicates a medium level of self-efficacy, and < 5 points indicates a low level of self-efficacy; the Cronbach’s *α* coefficient of the scale was 0.89.

The Simplified Geriatric Depression Scale (GDS-15) was first developed by Sheikh et al. (31) and consists of 15 items; 1 point is awarded for answering ‘no’ in questions 1, 5, 7, and 11, and 1 point is awarded for answering ‘yes’ in the remaining questions, totaling 15 points. The higher the score, the more severe the degree of depression. A score of 0 to 4 indicates no depression, 5 to 8 indicates mild depression, 9 to 11 indicates moderate depression, and 12 to15 indicates severe depressive symptoms. The scale has good reliability and validity, with a Cronbach’s *α* coefficient of 0.793. In our study, a depression score > 4 indicated that the patient had depression, while a score ≤ 4 indicated that the patient had no depression.

### 2.4 Data collection

**Figure 1:**
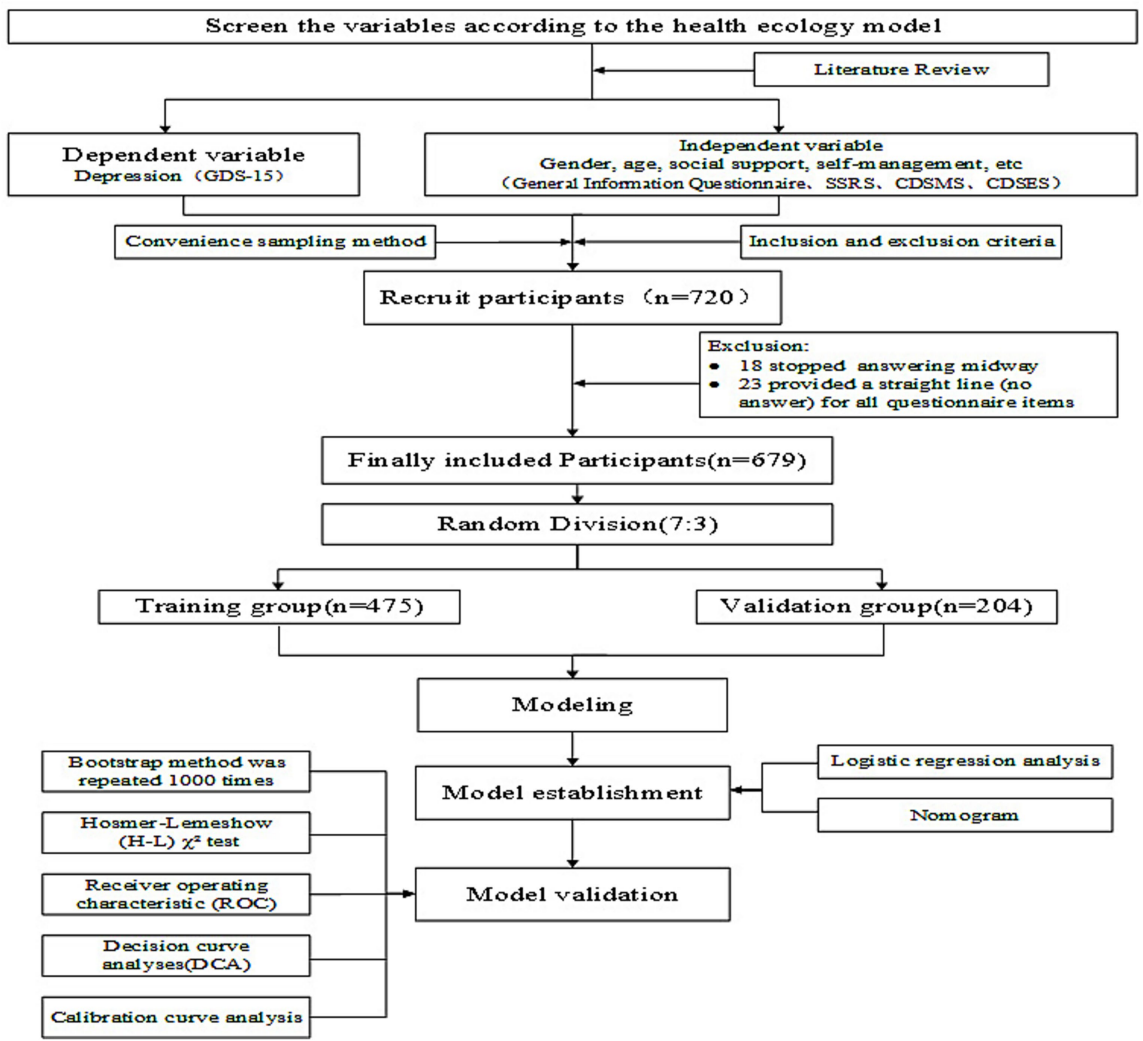
Flowchart describing the general framework of the study.

First, invitation letters were sent to the administrators of community health service institutions to secure their approval and cooperation. Ultimately, administrators from 12 community health service institutions responded and agreed to participate in the project. Second, following the project timeline, the research team sequentially screened older patients with chronic multi-morbidity who met the inclusion and exclusion criteria from residents with established health records in each community health service institution. Recruitment letters were then distributed to eligible patients, resulting in the enrollment of 720 community-dwelling older adults with chronic multimorbidity. After obtaining consent, the research team contacted each participant by telephone to schedule survey appointments and invited them to attend surveys conducted in meeting rooms at community health service institutions. Prior to the survey, investigators reiterated the purpose and significance of the study, along with relevant precautions, to all participants and requested them to sign informed consent forms. Participants capable of independently completing questionnaires did so autonomously, while those unable to self-complete were assisted through interviewer-administered question-and-answer formats. Investigators provided clarifications as needed, and all participants had the right to terminate their participation at any time. As shown in Figure 1. This research has been approved by the Ethics Committee of Ningxia Medical University (Review Number: Ningxia Medical University Ethics No. 2023–040).

### 2.5 Statistical analysis

A database was established using SPSS 25.0 for data processing and analysis. The statistical significance testing was two-sided with the test level *α* value of 0.05. Measurement data are presented as mean ± standard deviation, and t-test or one-way analysis of variance was used for comparison between groups. Numerical data are described as frequency (percentage), and the chi-squared test was used for comparisons between groups. The subjects were randomly assigned to the modeling group and the validation group at a ratio of 7:3 (32). Logistic regression analysis was employed to identify factors influencing depression in older adults with chronic multi-morbidity. The rms package in R software was used to draw the nomogram. The Bootstrap method was adopted to conduct 1,000 repeated samplings to complete the internal validation of the data, and the Hosmer-Lemeshow (H-L) χ^2^ test was used to evaluate the goodness of fit of the model. The discriminative ability and calibration of the prediction model were assessed using the area under ROC curve (AUC) and calibration curve analysis for both the modeling and validation groups. Clinical predictive utility was analyzed by decision curve analysis (DCA).

## 3 Results

### 3.1 Participant characteristics

Following recruitment and screening, a total of 720 patients participated in our survey, and 679 valid questionnaires were retrieved (18 participants stopped answering midway, and 23 participants provided a straight line (no answer) for all questionnaire items), with an effective recovery rate of 94.31%. Participant age ranged from 60 to 89 years old, with a mean age of 69.28 ± 6.71 years old. The proportions of males and females were 46.69 and 53.31%, respectively. The scores for social support, self-management, self-efficacy and depression were 32.49 ± 4.86, 21.70 ± 5.09, 6.43 ± 1.11 and 4.05 ± 2.91 points, respectively.

### 3.2 The incidence of depression among participants in the training group

The detection rate for depression among participants in the training group was 28.80%. Univariate analysis demonstrated that the detection rates of depression among older patients with multiple chronic diseases in the community with differing age, BMI, the number of chronic diseases, the frequency of weekly physical exercise, long-term regular medication use, types of medication, self-management, self-efficacy, social support, marital status, living status, educational level, occupation, and per capita monthly family income were all significantly different (*p* < 0.05; Table 2).

**Table 2:** Comparison of depression among participants with different characteristics in the training group.

Variable	Group	Total	No depression (*n* = 338)	Depression (*n* = 137)	*t/χ^2^*	*P*
Personal traits layer						
Age	60~	267 (56.21)	205 (60.65)	62 (45.26)	18.046	*<*0.001
	70~	168 (35.37)	115 (34.02)	53 (38.69)		
	80~	40 (8.42)	18 (5.33)	22 (16.05)		
Gender	Male	317 (46.69)	164 (48.52)	62 (45.26)	0.417	0.519
	Female	362 (53.31)	174 (51.48)	75 (54.74)		
BMI	<18.5 (Underweight)	23 (3.39)	6 (1.78)	7 (5.11)	12.415	0.006
	18.5 ~ <24.9 (Normal)	390 (57.44)	188 (55.62)	89 (64.96)		
	25 ~ <29.9 (Overweight)	223 (32.84)	126 (37.28)	31 (22.63)		
	30 ~ (Obesity)	43 (6.33)	18 (5.33)	10 (7.30)		
Family history	No	431 (63.48)	217 (64.20)	89 (64.96)	0.025	0.875
	Yes	248 (36.52)	121 (35.80)	48 (35.04)		
Number of chronic diseases	-	-	2.91 ± 1.02	3.14 ± 0.96	−3.125*	0.001
Behavior characteristics layer						
Smoking	No	521 (76.73)	259 (76.63)	100 (72.99)	1.574	0.455
	Yes	95 (13.99)	42 (12.43)	23 (16.79)		
	Quit smoking	63 (9.28)	37 (10.94)	14 (10.22)		
Drinking	No	582 (85.71)	284 (84.02)	120 (87.59)	0.976	0.323
	Yes	97 (14.29)	54 (15.98)	17 (12.41)		
Number of physical exercises per week	-	-	1.58 ± 0.50	1.39 ± 0.49	4.305*	*<*0.001
Regular medication	No	133 (19.59)	84 (24.85)	21 (15.33)	5.135	0.023
	Yes	546 (80.41)	254 (75.15)	116 (84.67)		
Type of medication	-	-	2.41 ± 1.53	3.21 ± 1.39	−3.559*	*<*0.001
Self-management	-	-	23.10 ± 5.05	20.14 ± 4.55	6.963*	*<*0.001
Self-efficacy	-	-	39.20 ± 6.37	32.02 ± 6.01	7.886*	*<*0.001
Interpersonal network layer						
Social support	**-**	-	33.84 ± 4.60	30.68 ± 4.97	5.181*	*<*0.001
Marital status	Married	554 (81.59)	285 (84.32)	100 (72.99)	8.144	0.004
	Unmarried/divorced/widowed	125 (18.41)	53 (15.68)	37 (27.01)		
Living conditions	Live alone	75 (11.04)	33 (9.76)	20 (14.60)	8.160	0.017
	Living with spouse	437 (64.36)	234 (69.23)	76 (55.47)		
	Live with children	167 (24.60)	71 (21.01)	41 (29.93)		
Living and working conditions layer						
Education	Primary school and below	252 (37.11)	99 (29.29)	67 (48.91)	20.786	*<*0.001
	Junior high school	201 (29.60)	104 (30.77)	41 (29.93)		
	Senior high school/junior college	192 (28.28)	113 (33.43)	25 (18.25)		
	Bachelor’s degree and above	34 (5.01)	22 (6.51)	4 (2.91)		
Working status	Employed	22 (3.24)	16 (4.73)	2 (1.46)	5.239	0.155
	Farming	194 (28.57)	82 (24.26)	39 (28.47)		
	Unemployed	59 (8.69)	27 (7.99)	12 (8.76)		
	Retirement	404 (59.50)	213 (63.02)	80 (58.40)		
Occupation	Worker	135 (19.88)	61 (18.05)	28 (20.44)	13.395	0.020
	Farmer	223 (32.84)	105 (31.07)	56 (40.88)		
	Enterprise or business unit personnel	169 (24.89)	95 (28.11)	27 (19.71)		
	Civil servants or managers	67 (9.87)	40 (11.83)	9 (6.57)		
	Businessman	23 (3.39)	11 (3.25)	1 (0.72)		
	Freelance/unemployed/others	62 (9.13)	26 (7.69)	16 (11.68)		
Address	Towns and cities	422 (62.15)	219 (64.79)	78 (56.93)	2.569	0.109
	Rural area	257 (37.85)	119 (35.21)	59 (43.07)		
Family per capita income	<2000	244 (35.94)	104 (30.77)	61 (44.53)	12.760	0.005
	≥2000 ~ 4,000	178 (26.22)	90 (26.63)	37 (27.01)		
	≥4,000 ~ 6,000	187 (27.54)	98 (28.99)	32 (23.36)		
	≥6,000	70 (10.31)	46 (13.61)	7 (5.10)		
Policy environment layer						
Medical payment methods	Basic medical insurance for urban employees	335 (49.34)	179 (52.95)	56 (40.88)	6.143	0.105
	Basic medical insurance for urban and rural residents	280 (41.23)	129 (38.17)	63 (45.98)		
	Self-funded	11 (1.62)	4 (1.18)	2 (1.46)		
	Others	53 (7.81)	46 (13.60)	16 (11.68)		

*Indicates *t*-value.

### 3.3 Multivariate analysis of depression in the modeling group

Patients in the training group were grouped according to whether they were depressed. First, we performed univariate analysis and variables with statistically significant differences were extracted. Then, binary logistic regression analysis was performed on these variables. Analysis showed that age ≥80 years, excess body weight, type of medication, self-management, self-efficacy, and education level of senior high school/college were key factors associated with depression in patients (all *p* < 0.05), as shown in Table 3.

**Table 3:** Results of binary logistic regression analysis of depression in older community patients with multiple chronic diseases.

Variable	*B*	Standard error	Wald *χ^2^*	*P*	OR	95% CI		VIF
						Upper limit	Lower limit	
(Constant)	8.454	1.771	22.784	*<*0.001	4692.586			-
Age 80~	1.091	0.464	5.520	0.019	2.978	1.198	7.400	1.242
BMI overweight	−1.402	0.687	4.167	0.041	0.246	0.064	0.946	1.074
Type of medication	0.428	0.134	10.186	0.001	1.535	1.180	1.996	1.822
Self-management	−0.080	0.029	7.423	0.006	0.923	0.871	0.978	1.264
Self-efficacy	−0.112	0.023	24.436	*<*0.001	0.894	0.856	0.935	1.278
Education			8.387	0.039				1.981
Education High school/Junior college	−1.296	0.464	7.806	0.005	0.274	0.110	0.679	-

CI, confidence interval.

### 3.4 Construction and verification of a risk prediction model for depression

**Figure 2:**
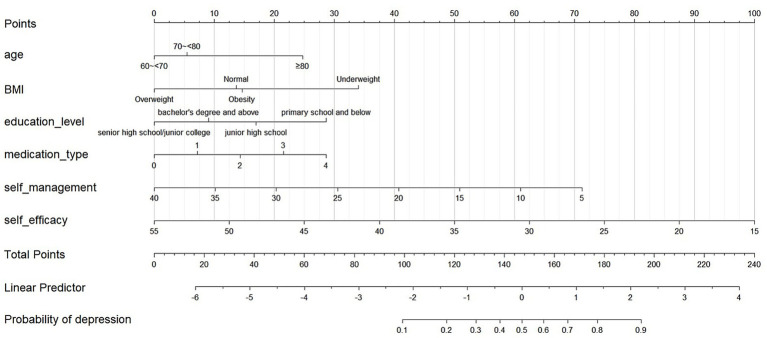
Nomogram for the risk of depression in older patients with multiple chronic diseases in the community.

**Figure 3:**
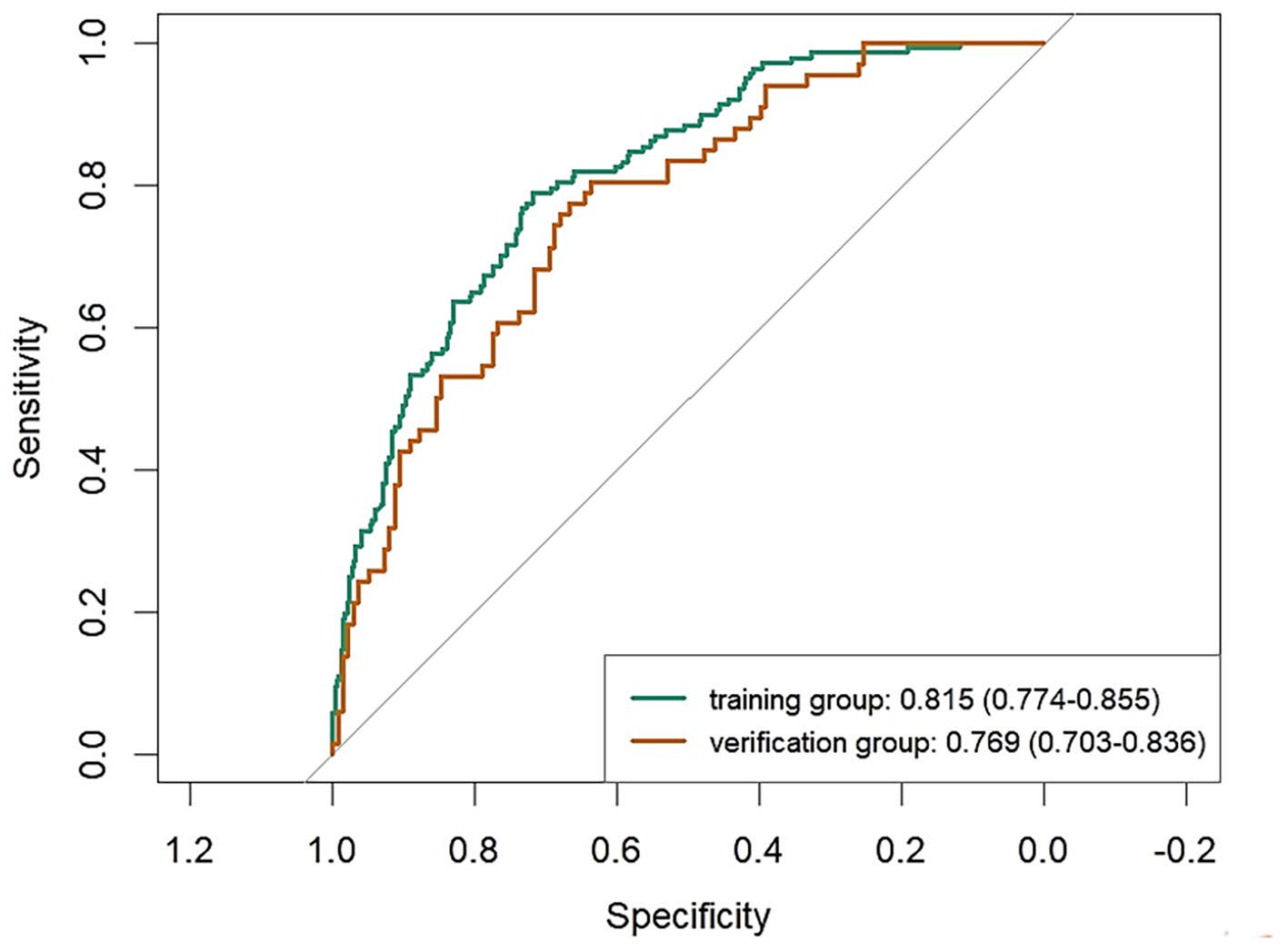
Area under the ROC curve for a predictive model for the risk of depression.

**Figure 4:**
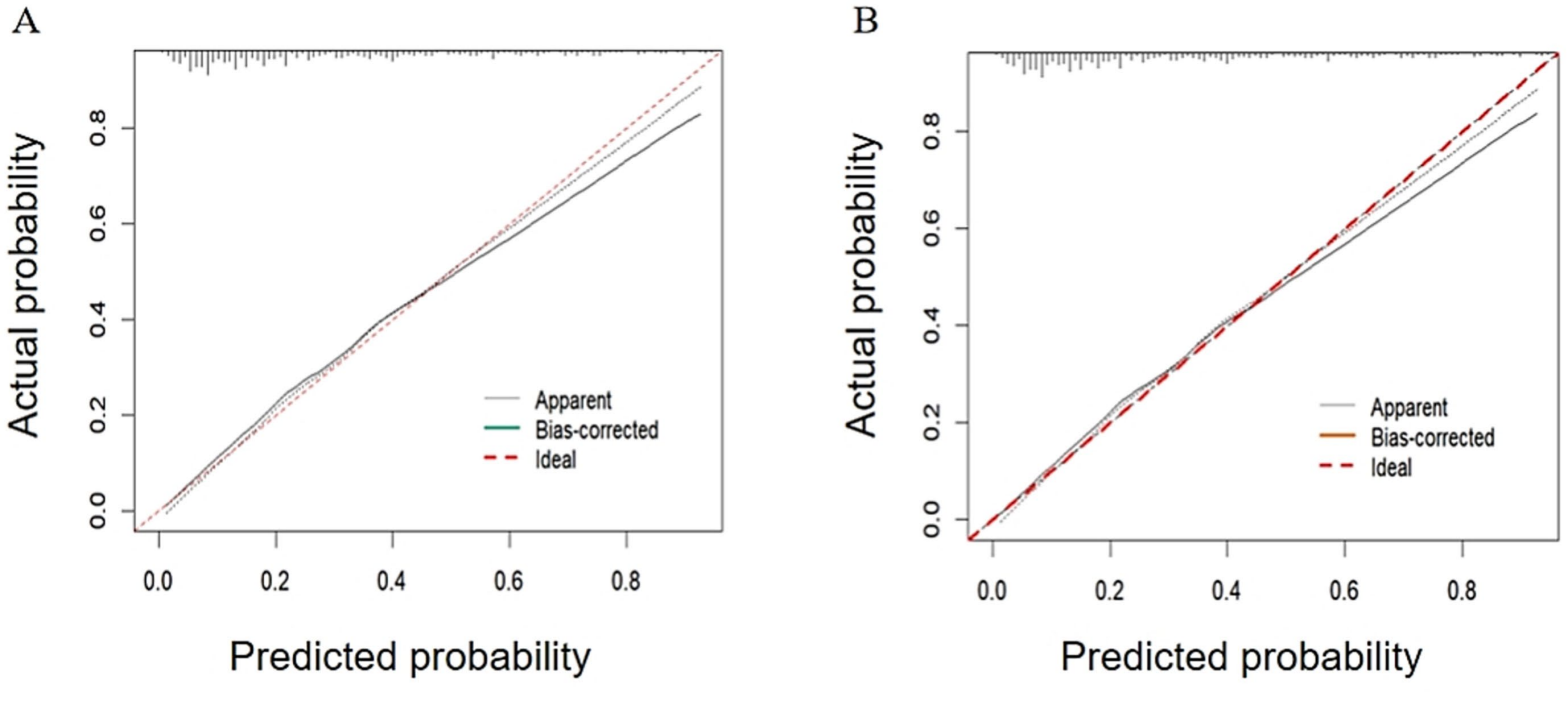
Diagram showing internal validation calibration for the new predictive model. Calibration curves for training group **(A)** and verification group **(B)**. The solid line represents the model after calibration. The closer the calibration curve of the model is to the ideal line, the better model’s predication accuracy.

**Figure 5:**
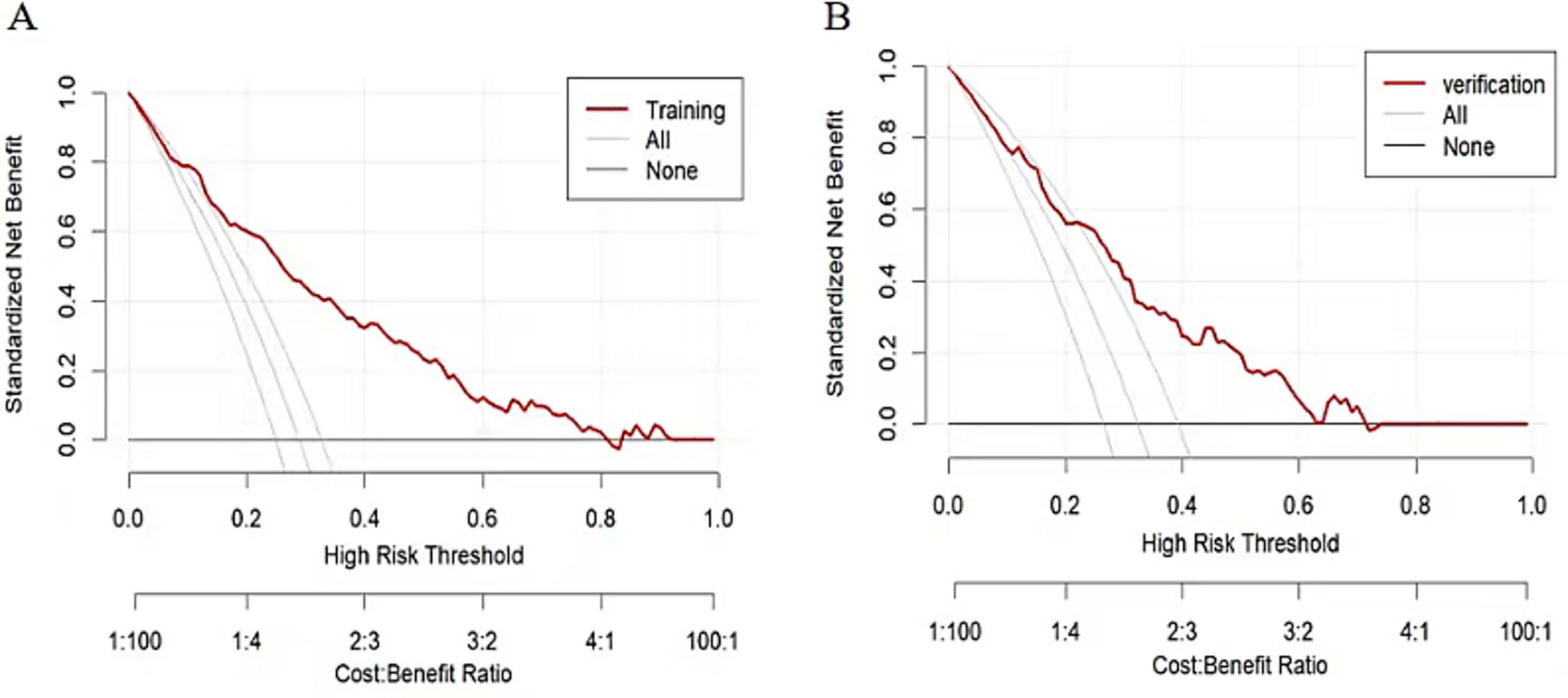
Model decision curve. Decision curve analysis (DCA) of the prediction nomogram. **(A)** training group. **(B)** verification group.

Following binary logistic regression screening, six statistically significant variables were identified. These variables were then used as the core to construct a prediction model (Figure 2). For internal validation, we used the Bootstrap method to perform 1,000 repeated sampling to complete the validation process. The area under ROC curve for the modeling and validation group was 0.815 (95% confidence interval [CI]: 0.774–0.885) and 0.769 (95% CI: 0.703–0.836), respectively (Figure 3). The DeLong test indicated no significant difference in the AUC of the ROC curves between the two groups (*Z* = 1.134, *p* = 0.258). The calibration graph (Figure 4) showed that the ability of the model to predict the risk of depression risk had a high degree of fit with the actual situation. The average absolute error between the predicted value and the actual value in the modeling group was 0.301; this compared to 0.343 in the validation group. These findings suggest that the model exhibited a moderate and acceptable level of error in predicting depression risk among community-dwelling older patients with multiple chronic diseases. The predictive accuracy of the nomogram model was further assessed using the Brier score. The Brier score was 0.15 for the training set and 0.19 for the validation set, indicating satisfactory probability prediction performance in both datasets. The mean squared error between the predicted probabilities and the observed occurrence of depression remained within an acceptable range. According to DCA analysis (Figure 5), when the threshold fell within the range of 0–1, the model curve was always above the reference line, thus indicating that the net benefit was better, thus indicating that the nomogram exhibited a good clinical effect. The accuracy, sensitivity and specificity of the model was 74, 79, and 72%, respectively. The Hosmer-Lemeshow fitting test resulted in a χ^2^ value of 8.80 (*p* = 0.359).

## 4 Discussion

To our knowledge, this is the first study to develop a nomogram-based risk prediction model for depression among older patients with chronic multimorbidity in communities of Northwest China, grounded in the health ecology model. This model incorporates multi-level factors spanning individual, interpersonal, and community dimensions, specifically addressing the complex high-risk population with multimorbidity. It thereby addresses limitations of existing prediction models, such as narrow theoretical frameworks and insufficient population specificity. Moreover, the nomogram-based risk tool provides community-based primary healthcare workers with a simple yet accurate method for individualized risk assessment, significantly enhancing early identification and preventive intervention capabilities. This study holds important theoretical and practical value for advancing early detection and precision management in geriatric mental health care.

Further analysis indicates that the nomogram developed in this study clarifies the mechanisms of multilevel risk factors. At the personal trait level, age and BMI function as fundamental physiological indicators, reflecting the potential influence of cumulative health decline and nutritional-metabolic status on mental health. At the behavioral characteristic level, self-management ability and self-efficacy represent modifiable protective factors, and their improvement may mitigate disease burden by enhancing individual control and coping confidence. In addition, an increased number of medications may indirectly affect psychological status through treatment complexity and medication burden. At the life and working condition level, educational attainment, serving as a proxy for socioeconomic status and cognitive resources, exhibited a protective effect, suggesting that education may influence depression risk through multiple pathways, including health literacy, social resources, and psychological resilience. A noteworthy methodological consideration is the indirect operationalization of macro-level ecological factors, such as community resource allocation and specific health policies, which was limited by data availability in this study. This approach may have led to an overestimation of the effects of individual-level predictors while underestimating the measurable impact of broader structural determinants. Future research should incorporate linked administrative, spatial, and policy data to develop multilevel models that more comprehensively capture the socio-ecological complexity of depression risk.

Different from the results reported by Silva Castro-de-Araujo et al. (33), our study demonstrated that the risk of depression among community-dwelling older patients aged 80 years and over with multiple chronic diseases was significantly increased, being 2.99 times higher than that among individuals aged 60–70 years (OR = 2.99, 95%CI: 1.20–7.40), Similar to the observations of Clare Tazzeo et al. (34), there are several possible explanations for this finding. First, advanced age is associated with reduced neurotransmitter regulatory capacity and cognitive decline. Concurrently, multi-morbidity often leads to pain and functional limitations, further elevating the risk of depression. Second, older adults are more likely to experience bereavement, reduced social engagement, and a loss of independence; these events can foster feelings of loneliness and diminished self-worth. Third, increased age may also intensify fears related to mortality and disease progression (35). Patients with comorbidities often harbor more pessimistic outlooks regarding their health, making them vulnerable to emotional distress and less capable of self-regulation. Therefore, healthcare providers should prioritize mental health support for the very old, incorporating interventions such as mindfulness-based therapy to alleviate physical discomfort and counter negative emotions, thereby helping to prevent or mitigate depression.

In agreement with Fleetwood et al. (36), our study found that the risk of depression among overweight community-dwelling older adults with chronic diseases was significantly lower than that among underweight individuals, being only 0.25 times as likely to develop depression (OR = 0.25, 95%CI: 0.06–0.95). One possible explanation for this is that adipose tissue secretes neurotransmitters that help to maintain emotional balance, whereas underweight individuals may experience hormonal imbalances that disrupt both metabolism and mood (37). Moreover, overweight patients often have better nutritional intake, which may enhance their ability to cope with illness. In contrast, an underweight status may reflect poor nutrition or disease severity, thus contributing to anxiety and helplessness. Community health workers should regularly monitor a patient’s weight, provide dietary guidance to those who are underweight, and promote balanced nutrition. Overweight patients should also receive dedicated education on healthy weight management to avoid obesity-related complications.

Polypharmacy is a common phenomenon among older adults with multiple chronic conditions. Our results, consistent with those of Han et al. (38), showed that the risk of depression increased with the number of medication types (OR = 1.54, 95%CI: 1.18–2.00), indicating that the risk of depression increased by 39% for each additional type of medication. This may be due to more severe illness, increased treatment burden, side effects, or financial strain. It is advisable for community health services to maintain follow-up records for patients on multiple medications, monitor disease progression, and reinforce adherence. When clinically appropriate, de-prescribing or switching to better-tolerated or insurance-covered drugs may help to reduce side effects and economic burden.

Supporting the findings of Schmitt et al. (39) and Roskoschinski et al. (18), we found that the risk of depression decreased by 8% for each 1 point increase in self-management score (OR = 0.92, 95%CI = 0.87–0.98). In addition, the risk of depression decreased by 11% for each 1 point increase in self-efficacy score (OR = 0.89, 95%CI = 0.86–0.94), indicating that higher self-management and self-efficacy scores were negatively associated with depression among community-dwelling older patients with chronic diseases. Self-management entails the set of skills and behaviors that patients utilize to manage their health conditions effectively (40), while self-efficacy reflects confidence in handling health-related challenges (41). Higher levels of both self-management and self-efficacy are associated with better disease coping skills, proactive health behaviors, and positive psychological adjustment, thereby reducing the risk of depression.

Our analyses also identified educational attainment as a significant factor associated with depression risk. Consistent with Ha et al. (42), a high school/college education level was a key protective factor for depression among community-dwelling older patients with multiple chronic diseases (OR = 0.27, 95%CI = 0.11–0.68). The risk of depression in individuals with a high school/college education level was 0.27 times that of those with a primary school education or below, which may be related to better health literacy and improved access to health information. Such individuals may be more capable of adopting scientific approaches to chronic disease management and regulating physical and psychological stress. This core protective effect was also reflected in the relatively low score weight assigned to this variable in the nomogram. Participants with a junior high school education (OR = 0.58, 95%CI: 0.33–1.02) and those with a bachelor’s degree or above (OR = 0.41, 95%CI: 0.12–1.38) exhibited a reduced risk of depression; however, both 95%CI included 1, indicating a lack of statistical significance and suggesting that these findings should be interpreted as exploratory. The non-significant protective effect observed for junior high school education may be attributable to the limited improvement in health literacy at this level. For participants with a bachelor’s degree or above, no significant protective effect was observed, which is inconsistent with some previous studies and may be primarily due to the small sample size of this subgroup (*n* = 34), resulting in insufficient statistical power and unstable estimates. In addition, we propose the following exploratory hypotheses for future investigation: older adults with higher educational attainment may have elevated expectations regarding their own health, and the gap between expectations and reality when confronted with multimorbidity and functional decline may partially offset the cognitive and social resource advantages typically associated with higher education. At the same time, potential confounding factors, such as the long-term effects of occupational stress and the complexity of social role transitions, may not have been adequately measured in this population. Therefore, the precise relationship between having a bachelor’s degree or above and depression risk warrants further validation in larger samples.

The risk prediction model in our study is presented in the form of a nomogram, based on the five dimensions of the HEM, after summarizing and integrating variables. Six predictors were selected by binary logistic regression. Our model will allow clinicians to estimate an individual’s risk of depression by summing scores assigned to each predictor, thus facilitating a shift from reactive care to proactive prevention. Internal validation using bootstrapping (1,000 samples) showed good discriminative ability, with AUCs of 0.815 (for the modeling set) and 0.769 (the validation set). Calibration curve analysis further confirmed the accurate prediction of outcomes, as evidenced by the close fit to the observed data. The model achieved an accuracy, sensitivity and specificity of 74, 79 and 72%, respectively. Assessment using decision curves further verified the usefulness of our model in screening scenarios.

## 5 Limitations

Our study has several limitations that warrant consideration. First, all participants were recruited from a single city in Northwest China. Although this approach ensured sample homogeneity and facilitated data quality control, it restricts the geographical representativeness and external validity of the findings. Regional differences in healthcare resource allocation, medical insurance policies, and social environments may influence the generalizability of environment-related variables included in the model. Future studies should perform multicenter external validation across diverse geographical regions to evaluate the broader applicability of the model.

Second, to maintain a clear research focus, the analysis was limited to 10 chronic conditions selected on the basis of their prevalence and common comorbidity patterns among the older population in China. Because not all possible disease combinations and their interactions were included, the predictive accuracy of the model may be limited for older adults with other comorbidity profiles. Future research could incorporate more comprehensive disease assessment indicators to better reflect the multidimensional burden of chronic diseases.

Third, due to constraints associated with field investigations, information on non-respondents was not recorded, and no comparative analysis between respondents and non-respondents was conducted. Therefore, potential selection bias cannot be entirely excluded, which may affect sample representativeness and external validity. Caution is required when generalizing the study conclusions.

Fourth, owing to practical and ethical considerations in community-based surveys, physiological indicators related to neurological and immune function, such as serotonin or C-reactive protein levels, were not collected. Although this does not compromise the practical utility of the model in community settings, it limits the inclusion of core biological variables that may help explain the comorbidity between chronic diseases and depression. Objective physiological markers could provide additional predictive information independent of psychosocial factors and contribute to elucidating the underlying mechanisms of depression. Future studies may integrate such biomarkers with multidimensional psychosocial variables to develop models with stronger explanatory capacity and greater precision for guiding bio-psychosocial interventions.

Fifth, the use of logistic regression to construct the risk prediction model restricts its capacity to handle high-dimensional data and may increase susceptibility to multicollinearity. The model may also be less robust to data bias and outliers, and its predictive performance and generalizability may be inferior to those of certain machine learning approaches. In addition, the potential nonlinear relationship between age and depression was not fully assessed in this model, which may have led to the omission of important age-related effects. Future research could explore hybrid modeling strategies that combine the predictive strength of machine learning algorithms with the interpretability of logistic regression to further enhance predictive performance.

Finally, this study conducted internal validation using the Bootstrap method with 1,000 repeated samples but did not perform external validation. This limitation constrains the assessment of the model’s predictive stability and accuracy across different regions, medical institutions, and heterogeneous older populations with comorbid conditions. Future studies should undertake multicenter external validation to further evaluate the generalizability and stability of the model in diverse populations.

## 6 Conclusion

Here, we used the HEM as core support to construct a predictive model for the risk of depression for older patients with comorbidities residing in the community. Our analyses showed that the risk of depression in this population was moderate, and was influenced by individual, interpersonal and social factors. Our new model achieved an accuracy, sensitivity and specificity of 74, 79 and 72%, respectively, thus indicating good prediction performance. This model can help the community to accurately identify high-risk groups, reducing the probability of depression by multi-dimensional intervention, and provide practical reference for the health management of older patients with comorbidities and community comprehensive management.

## Data Availability

The raw data supporting the conclusions of this article will be made available by the authors, without undue reservation.
